# What’s Happening During Home Visits? Exploring the Relationship of Home Visiting Content and Dosage to Parenting Outcomes

**DOI:** 10.1007/s10995-018-2547-5

**Published:** 2018-06-13

**Authors:** Peggy Nygren, Beth Green, Katie Winters, Anna Rockhill

**Affiliations:** 1 Regional Research Institute for Human Services, Graduate School of Social Work, Portland State University, Market Center Building, Suite 900,1600 SW 4th Ave, Portland, OR 97201 USA; 2 Center for Improvement of Child and Family Services, Graduate School of Social Work, Portland State University, Market Center Building, Suite 400,1600 SW 4th Ave, Portland, OR 97201 USA

**Keywords:** Early childhood home visiting, Home visiting program content, Home visiting program dosage, Family risk factors, Maternal risk factors, Parenting outcomes

## Abstract

*Introduction* Research has documented modest positive impacts of early childhood home visiting programs. However, understanding more about what home visitors do during visits and how much time they spend on specific topics may provide insight into the variability in effectiveness of services. *Methods* Outcome data were collected via parent survey at program enrollment and 12 months from 123 women in three MIECHV-funded home visiting models. Home visitors completed weekly home visit content and activity logs. *Results* Families received an average of 28 visits during the study (3.1 visits per month). Of ten content areas, the three most often discussed were early childhood development, physical care of children, and the parent–child-relationship. Multivariate regression models were used to explore the association of home visit dosage, home visit content and cumulative risk factors on parenting outcomes. Women whose visits were focused more on parenting topics reported lower parenting-related stress at follow-up compared to those whose visits had less parenting content. Additionally, higher-risk women who received greater numbers of home visits showed larger reductions in their attitudes about harsh punishment over time, compared to high-risk women with fewer home visits. *Discussion* Receiving home visits that emphasize parenting content may contribute to reduced parenting-related stress. For high-risk women in particular, receiving more visits overall may be important to achieving positive outcomes. Implications for practice include working to engage and retain high-risk families. Future home visiting research calls for improved methods for collecting data on content/activity during visits, the necessity for long-term follow-up, and testing for the effectiveness of varied and flexible visit schedules/content focus for women and families with trauma exposure.

## Significance

While research has documented small-to-modest positive program impacts of early childhood home visiting in various models, less is known about how home visit dosage and the specific content covered during home visits influence parenting and family outcomes. Wide variations across home visiting models are found in both how many visits women receive and the type of activity during visits. Many of the women screened eligible for home visiting services are currently experiencing multiple life challenges and/or have significant trauma histories. For women and families with multiple risk characteristics, understanding how dosage and content relates to parenting outcomes is critical to improving program effectiveness and to guiding program and home visitor practices. Our work sought to address these gaps by linking detailed information about the dosage (# of visits) and content areas of home visits and risk characteristics of participants, to parenting outcomes including stress, knowledge, and attitudes.

## Introduction: Research on Home Visiting Service Delivery

While research focused on the nature and content of home visiting remains sparse, a recent meta-analysis found that effect sizes for numerous program outcomes varied depending on program structure and approach (Filene 2012; Filene et al. 2013). Specifically, program-related variables such as having professional vs. nonprofessional home visitors, matching home visitors and women based on race/ethnicity, and relative emphasis on various topics such as parenting and responsiveness were associated with positive effects on some outcomes (Filene 2012; Filene et al. 2013). Utilizing descriptions of program models and curricula, the authors found larger effects in parenting-related outcomes for programs that emphasized information about developmental expectations and specific behavior management skills. One study that collected data specific to visit content reported that the larger the percentage of time home visitors spent on child-focused activities, the greater positive outcomes were found for child cognitive and language development, parenting, and maternal depression (Raikes et al. 2006).

Meta-analytic strategies to summarize home visiting outcome literature looking at service variability, including dosage predictors, point to increases in number of total hours in home visits, and home visit ‘frequency/intensity’ to be related to stronger program effects (Sweet and Appelbaum 2004; Nievar et al. 2010). Research examining how home visiting dosage and content influence outcomes is complicated both in terms of how different researchers operationalize “dosage” and by the interaction between level of family risk and service delivery. Research contends that as the number of risk factors accumulate for women and families, so does the potential for negative maternal and child outcomes (Burchinal et al. 2008; Trentacosta et al. 2008; Cabrera et al. 2011). At the same time, families at highest risk for negative outcomes and who may be most in need of services, may be challenging to both enroll and retain in services (Gomby et al. 1999; Howard and Brooks-Gunn 2009).

To address these gaps, the following exploratory research questions were developed:

1. What content areas comprise the time spent in MIECHV-funded home visits?
2. Do women who receive more home visits report greater improvement in parenting attitudes, knowledge, or parenting stress?
3. Do women whose visits are characterized by a greater emphasis on parenting content show more improvement in parenting-related outcomes?
4. Does the influence of number of home visits on outcomes differ for families at higher-risk for negative family outcomes compared to those at lower-risk?

## Methods

Research was conducted in accordance with ethical principles and guidelines, and reviewed and approved by the Oregon Health Authority, Public Health Division, Institutional Review Board.

### Study Recruitment

Study participants were newly enrolled or within 6 visits in MIECHV funded home visiting services in 13 counties in Oregon. Women were 16 years of age or older, spoke either English or Spanish, and either pregnant or parenting a child < 12 months of age. Home visitors asked interested women for their consent to be contacted by the research team, who then sent study information and the baseline survey via either mail or email. Participants also provided consent for their home visitor to provide the research team with regular information about their visits. For clarity, the terms women and participants will refer to those who consented to be in the study.

### Data Collection

Participating women completed surveys at study enrollment (baseline) and again 12 months later. Women received a $25 gift card incentive to a local store for completing the Time 1 survey and a $40 gift card at Time 2. Research staff contacted participants monthly between Time 1 and Time 2 to confirm their contact information and support study retention. In all, 132 out of 197 women who expressed initial interest in participating in the study completed a Time 1 survey (67%) and were included in the study. We do not have systematic data on those who chose not to complete the baseline survey, however, some were not eligible due to recruitment window parameters or stated exclusion criteria. Of the 132 Time 1 respondents, 123 (94%) returned a Time 2 survey. Forty-five home visitors working with women provided weekly logs detailing home visiting content. Approximately 90% of expected weekly logs were submitted, with an average of 32.6 logs per family (range 1–60).

#### Measures: Participant Surveys

Baseline surveys included demographic, and individual and family risk information. Risk factors were identified based on known correlates of negative parenting behaviors, harsh punishment, or extreme parenting stress, selecting brief, validated screening tools whenever possible. In some cases, we worked with state home visiting partners to shorten existing measures to reduce burden to participants. Indicators of psychosocial risk level were: Low social support (the number of people women could turn to for support); Presence of family relationship problems (“none or minor”, “some”, or “serious”); Depression risk (PHQ-9; Kroenke et al. 2001); Presence of interpersonal family violence (Pregnancy Risk Assessment Monitoring System-Phase 6; Centers for Disease Control and Prevention 2009); Maternal substance use (3-item version of the Simple Screening Instrument for Substance Abuse (SSI-SA); Knight et al. 2000); and history of adverse experiences (4-item version of the Adverse Childhood Experiences Questionnaire; Centers for Disease Control and Prevention 2014).

The items on the SSI-SA included three questions asking about drug use and problems related to drugs or alcohol in the past 6 months, and a fourth question about having a current drinking or drug problem. For adverse experiences growing up, respondents indicated if they had ever been in foster care, or if anyone in their family had a problem with drugs or alcohol abuse, depression or mental health issues, or incarceration. We chose not to ask questions about participants’ experience of maltreatment within their family of origin (a known risk factor for negative parenting), given the intrusiveness of these questions in terms of potential for retraumatization and lack of face-to-face support during survey administration. Each indicator was dichotomized to indicate the presence of the risk factor (1 = yes, 0 = no).

#### Cumulative Risk Factor Index

A cumulative risk factor index was calculated using the sum of 12 dichotomized risk variables including: becoming a mother at 19 or younger, premature birth of their child, less than a high school education, housing instability, household unemployment, single relationship status, low social support, troubled relationships, depression, interpersonal violence, drug problems, and adverse childhood experiences. The substance abuse problem items were dichotomized such that if a mother indicated a positive response to any items (e.g., had used too much, tried to cut down, or felt like she had a drug problem), it was coded as the presence of the drug problems risk factor.

#### Outcome Measures

Parenting outcomes were collected at Time 1 and Time 2. *Parenting knowledge* was assessed with the UpStart Parent Survey (USPS) Parenting Knowledge/Skills subscale (Benzies et al. 2013). *Parenting attitudes* were assessed using Corporal Punishment and Empathy subscales from the Adult Adolescent Parenting Inventory (AAPI-2; Bavolek and Keene 2001). We also used two of three subscales of the Parenting Stress Index-Short Form (PSI-SF), the Parenting Distress (PD) and Parent–Child Dysfunctional Interaction (P-CDI) subscales (Abidin 1995; Haskett et al. 2006), to measure stress related to the parenting role. See Table 1 for example items for measures and reliability data.

**Table 1:** Parenting outcomes: example of items on measures

**Measure domain** Name of measure *Sub-scale*	Cronbach’s Alpha	# of Items	Example items
**Parenting attitudes**			
Adult Adolescent Parenting Inventory (AAPI-2)	.793	14	
*Corporal Punishment subscale*	.845	8	1. Children can learn good discipline without being spanked 2. A good spanking lets children know that parents mean business
*Empathy subscale*	.587	6	1. The sooner children learn to feed and dress themselves and use the toilet, the better off they will be as adults 2. Children should know what their parents need without being told
**Parenting stress**			
Parenting Stress Index (PSI-SF)	.881	24	
*Parenting Distress (PD) subscale*	.856	12	1. Since having a child, I feel that I am almost never able to do things that I like to do 2. I feel trapped by my responsibilities as a parent
*Parent–Child Dysfunctional Interaction (P-CDI) subscale*	.847	12	1. My child rarely does things for me that make me feel good 2. My child doesn’t seem to learn as quickly as most children
**Parenting knowledge**			
UpStart Parent Survey (USPS)	.682	10	
*Parenting Knowledge*/Skills	N/A	2	1. I know how to set clear limits for my child/children 2. I know how to keep my child/children safe

#### Number of Home Visits

Given the variability in the timeframes when home visit logs were collected, we used home visit data housed in the MIECHV Oregon administrative database for home visit total dosage. The program dosage outcome was calculated as the *total number of visits* received by participants between their enrollment date and the date they completed the Time 2 survey. We also used this strategy due to concerns that the amount of time spent in home visits may have reflected program requirements rather than actual time spent.

#### Home Visit Content

The content log was developed based on a thorough examination of the literature, review of existing tools (Home Visit Rating Scales; Boller et al. 2009), and in consultation with home visiting research experts and stakeholders. We also incorporated home visiting service areas from the Mother and Infant Home Visiting Program Evaluation study (U.S. Department of Health and Human Services Administration for Children and Families Office of Planning, Research and Evaluation 2015). Content areas were refined based on feedback from home visiting model leads and home visitors about typical visit topics and seemed to have good validity; however, we did not systematically validate this measure. Home visitors accessed an on-line log system to document the estimated time spent during visits in ten specific content areas (see Table 2). Incremental time spent response categories were developed due to the reported difficulty of home visiting staff, and potential inaccuracy, of estimating *actual time* spent. Response choices for content areas included “did not discuss”, “touched on briefly”, “discussed 10–15 min” and “discussed more than 10–15 min”. Logs were to be completed after each home visit and submitted electronically to the research team, including reporting when no visit occurred for the week.

**Table 2:** Home visiting content areas and activity log examples

Content area	Examples within content area
Taking care of self: physical health	Prenatal health, nutrition, exercise, substance use, smoking
Taking care of self: emotional health	Maternal mental health, stress, coping, well-being
Taking care of self: relationships	Communication, relationship with partner, domestic violence
Parenting: child physical care	Physical care of child, breast feeding/nutrition, home safety
Parenting: parent–child relationships	Attachment, responsiveness, reciprocity, affection, empathy
Parenting: early childhood development	Temperament, development (social/physical), appropriate expectations
Parenting: guidance	Modeling, positive discipline, behavior management, routines
Life course	Goal setting, family planning, education, employment
Support networks: caregiver support	Social/parent support, childcare, father involvement, parenting classes
Support networks: information/referrals	Emergency/crisis plans, housing, utilities, TANF/SNAP/OHP

*TANF* temporary aid to needy families, *SNAP* supplemental nutrition assistance program, *OHP* Oregon health plan

Because there were more actual visits documented in the MIECHV database when compared to number of logs received, we elected to create an overall estimate of time spent on each area across all logs received for a family. First, study content data were collapsed into the four overall topics or domains with similar conceptual focus: self-care, parenting, life course, and support networks/referrals. This average rating was then multiplied by the number of home visits (from MIECHV database) the family received to generate the estimated “*content dosage*”. Thus, the content dosage variable does not reflect actual time estimates, but provides a proportional representation of the amount of time on a given domain across all visits. For example, two families with a similar average amount of time spent on self-care across home visits, but who had different quantities of home visits, would have different “content dosage” scores for self-care.

### Analysis

Multivariate regression models were tested using Time 2 outcome scores for each of the primary parenting outcomes (UpStart, AAPI, and PSI), controlling for scores at Time 1. All models included the following covariates: white/non-white, completed high school (yes/no), marital status, number of adverse childhood experiences (ACEs), and depression risk. To examine whether home visit dosage had differential effects on outcomes for women with higher-risk versus lower-risk profiles, we used the cumulative risk score to calculate multiplicative interaction terms (e.g., number of visits × cumulative risk score) and included terms in the models as predictors. For models testing interaction effects, demographic characteristics included in the cumulative risk score were not included as covariates.

## Results

### Study Sample and Descriptive Data

Table 3 provides demographic characteristics for women in the study. Slightly more than half of the women reported White race, while 21% reported Hispanic/Latina race/ethnicity.

**Table 3:** Selected study participant demographic characteristics

Baseline demographic & risk measures	% or mean	N
Women		
Pregnant at enrollment	41.0%	122
Number of children (mean)	1.5	121
Age (mean)	25.5	121
Race/ethnicity^a^		
White	53.7%	123
Hispanic/Latina Origin	21.1%	123
Multi-racial	17.9%	123
Black	4.1%	123
American Indian	1.6%	123
Hawaiian/Pac. Islander	0.8%	123
Homeless in the last year	8.9%	123
Most of the time, trouble paying basic expenses	24.4%	123
More than minor relationship problems	30.9%	123
Depression; moderate or severe	20.5%	122

^a^20% were categorized as ‘other’; 0% Asian

Women had an average of 3.4 of a possible 12 risk factors (range 0–8 of possible 12; SD 1.98). Sixty-one percent of women had between 1 and 4 risk factors, and 26% of women reported between 5 and 8. Only 7% of women had zero risk factors. Descriptive information on parenting outcome scores is presented in Table 4 for both the baseline and 12 month surveys.

**Table 4:** Parenting outcome scores at Time 1 (baseline) and Time 2 (follow-up)

Parenting outcome measure	*Time 1* Mean (SD)	*Time 2* Mean (SD)
AAPI total score (n = 109)	1.74 (0.45)	1.69 (0.48)
AAPI Corporal Punishment subscale (n = 121)	1.98 (0.68)	1.89 (0.71)
AAPI Empathy subscale (n = 121)	1.43 (0.35)	1.42 (0.41)
UpStart (n = 84)	− 0.77 (0.55)	− 0.05 (0.53)
Parenting Stress Index (n = 85)	42.55 (12.0)	40.82 (12.7)
PSI Dysfunctional Interaction subscale (n = 86)	16.71 (5.0)	16.45 (5.3)
PSI Distress/Stress subscale (n = 85)	25.81 (8.7)	24.32 (9.2)

Table 5 presents descriptive information on the time spent in specific home visiting content areas as reported by home visitors on weekly logs. Home visit logs indicated a stronger focus on providing parenting information, with more than half of reported visits spending “at least 10–15 min” on early childhood development, physical care of children, or the parent–child-relationship. On average, the least time was spent on information and family resource referrals, with about a quarter of visits not covering resources at all, and 61% covering the domain only briefly. Considerable visit time was devoted to maternal self-care, especially maternal emotional health, with 45.7% of visits spending at least 10–15 min on the mental health of the mother.

**Table 5:** Average time spent in content area reported by home visitors on weekly logs

Content area	Estimated average time spent per family			
	None (0) %	Briefly (1) %	At least 10–15 min (2) %	More than 15 min (3) %
Taking care of self				
Physical health	14.7	50.0	32.8	2.6
Emotional health	0.9	51.7	45.7	2.6
Relationships	12.1	64.7	23.3	0.0
Parenting				
Physical care	3.4	30.2	57.8	8.6
Parent–child relationship	0.0	38.8	56.9	4.3
Early childhood development	1.7	22.4	66.4	9.5
Guidance	10.3	58.6	30.2	0.9
Life course				
Goal setting, planning	1.7	59.5	36.2	2.6
Support network and referrals				
Caregiver support	8.6	63.8	26.7	0.9
Information/referrals	26.7	61.2	12.1	0.0

Table 6 details program dosage based on number of home visits, as well as the average content dosage in four domains. The data indicate considerable variability in the number of home visits families received, ranging from 1 to 56 visits (mean 28 visits; SD 14.9). Visits were approximately one hour, on average (mean 67.46 min; SD 11.4; range 40–98). Proportionately, a greater amount of time in visits was spent on the parenting content domain compared to the self-care, life course, or support network/referrals content domains.

**Table 6:** Time spent in home visiting (dosage) and content dosage (four domains)

Home visit variables: dosage and content dosage	Mean (SD)	Min	Max
Average number of home visits received (n = 107)	28.21 (14.89)	1	56
Average length of home visits, minutes (n = 103)	67.46 (11.39)	40	98.18
Average estimated content dosage for self care^a^ (n = 111)	34.6 (18.9)	2.44	93.1
Average estimated content dosage for parenting^a^ (n = 111)	45.3 (26.5)	0.5	136.19
Average estimated content dosage for life course^a^ (n = 111)	37.7 (24.0)	0	112.0
Average estimated content dosage for support network/referrals^a^ (n = 111)	27.2 (16.7)	2	87.75

^a^Estimated dosage is calculated by weighting the average amount of time spent per content domain by the number of home visits received by the family

### Association of Dosage and Content Dosage to Parenting Outcomes

Separate regression models were tested for each of the three parenting outcomes, with predictors modeled separately for each. Predictors included dosage (number of visits), and content dosage in self-care, parenting, life course, and support network/referrals. All models included the covariates described previously. Results of regression models are shown in Table 7.

**Table 7:** Regression model results—association of home visiting estimated content dosage to Time 2 outcomes, controlling for Time 1 status

Outcome	Standardized beta (B)	T	Sig.
AAPI			
1. Total number of home visits	− .083	− 1.10	.274
2. Estimated dosage of self-care	− .043	− .560	.577
3. Estimated dosage of parenting	− .051	− .658	.512
4. Estimated dosage of life course	− .032	− .416	.679
5. Estimated dosage of support network/referrals	− .052	− .667	.506
Parenting Stress Index			
1. Total number of home visits	− .113	− 1.120	.267
2. Estimated dosage of self-care	− .177	− 1.817	.074^†^
3. Estimated dosage of parenting	− .249	− 2.632	.011*
4. Estimated dosage of life course	− .170	− 1.742	.086^†^
5. Estimated dosage of support network/referrals	− .172	− 1.733	.088^†^
UpStart			
1. Total number of home visits	.007	.069	.946
2. Estimated dosage of self-care	.161	1.586	.118
3. Estimated dosage of parenting	.076	.740	.462
4. Estimated dosage of life course	.116	1.131	.262
5. Estimated dosage of support network/referrals	.142	1.378	.173

Regression coefficients represent the effect of each of five dosage predictors [number of visits received (1) and type of home visit content (2–5)] on Time 2 outcomes controlling for Time 1 outcomes and for the following covariates: white/non-white, high school education, married/partnered, total depression score (PHQ scale), total number of adverse childhood experiences

^†^p ≤ .10; *p < .05; **p < .01

**Fig. 1:**
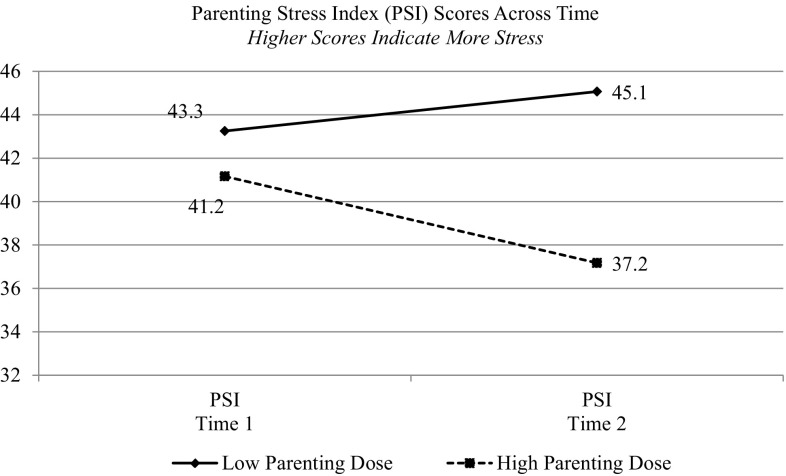
Higher parenting related content dosage is related to lower parenting stress at Time 2 for home visited families

Parenting content dosage was significantly associated with decreased parenting stress at Time 2. We identified a trend toward significance, indicating an association in which other content areas predicted parenting stress as well, although the number of visits alone was not related to decreased stress. Figure 1 displays the association between parenting content dosage and parenting stress, categorizing parents as receiving “high” versus “low” parenting content dosage (using a median split for high/low). Neither the number of visits nor the four content dosage areas were associated with changes in parenting attitudes (AAPI) or parenting knowledge (UpStart).

### Association of Family Risk Factors and Home Visit Dosage to Parenting Outcomes

**Fig. 2:**
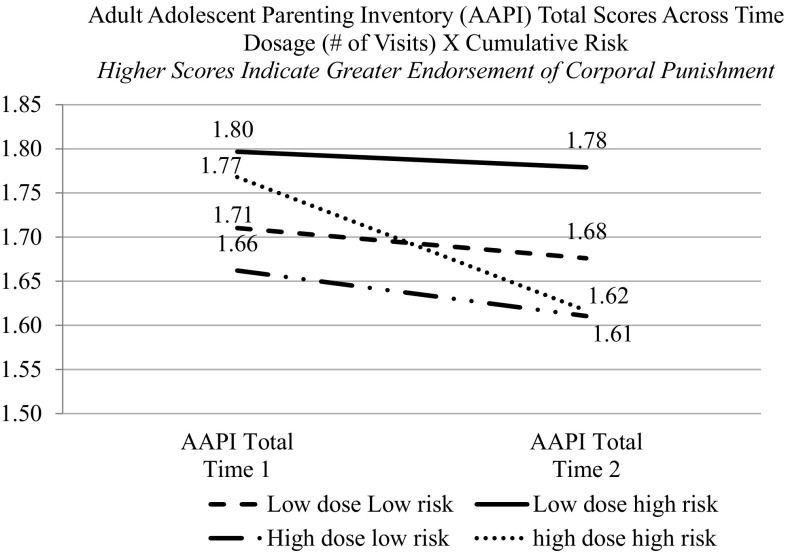
Cumulative risk moderates the impact of dosage (# of home visits) on attitudes toward corporal punishment for home visited families

The final research question explored whether the effects of dosage on outcomes differed for families with varying risk levels. Regression models used the Time 2 parenting outcomes and demographic covariates as noted above, and also included the following predictors: outcome scores at Time 1, cumulative risk factor index score, home visit dosage (number of home visits), and the cumulative Risk Factor Index × Home Visit Dosage interaction (Table 8). A significant main effect of risk was found, such that those families with more risk factors were more likely to endorse the use of corporal punishment. The interaction, Risk Factor Index × Home Visit Dosage, was also significant (Fig. 2). The interaction suggests that attitudes towards corporal punishment (i.e., indicating less positive attitudes towards corporal punishment with lower scores at T2) improved more for families who were higher-risk but also received a greater number of home visits compared to higher-risk families who received a low number of visits. In post hoc tests looking at the differences between the T1 and T2 scores of the AAPI, for the Corporal Punishment subscale, only the high-risk, high dosage participants showed a significant change (reduction in endorsement of harsh parenting practices). In this instance, the AAPI Empathy subscale was not a key driver in explaining results.

**Table 8:** Regression models testing moderating effect of risk factor index (# of risks) × dosage (# of home visits) on parenting outcome effects

Outcome	Standardized beta (B)	t	Sig.
AAPI (higher = greater endorsement of harsh parenting practices)			
Number of risks	.411	2.453	.016*
Risk by number of home visits interaction	− .472	− 2.423	.017*
PSI (higher = more stress)			
Number of risks	.253	1.078	.285
Risk by number of home visits interaction	− .270	− 1.068	.289
UpStart (higher = greater knowledge)			
Number of risks	− .209	0.803	.425
Risk by number of home visits interaction	.354	1.223	.225

^†^p ≤ .10; *p < .05; **p < .01

## Discussion

The current study focused on the relative emphasis on a variety of topics across the span of home visits received by families for up to 1 year. While visit content varies considerably, we found a relatively greater emphasis on parenting-related content areas. In particular, home visits were most likely to focus on information related to the physical health of the child, child development, and support for the parent–child relationship, all critically important during the child’s earliest years of life. The prominence on parenting and child development-related topics is not surprising, given the emphasis of the three home visiting models studied, all of which aim to improve parenting skills and support strong parent–child relationships. Also worth noting is the finding that home visitors dedicated a substantial amount of time, on average, to helping women take care of their own physical and emotional health, maternal self-care, and supporting family stability and adult life goals as compared to other areas. Relative to content related to life goals, resource referrals, and broader network supports, women spent more time talking with home visitors about their own emotional health. The focus on women’s mental health needs may reflect the growing awareness in the home visiting field of issues related to maternal depression, and the need to provide trauma-informed services to women who may have experienced one or many adverse life experiences. Within MIECHV programs, depression screening and referral is a required element of services, and working with women with depressive symptomatology has been an area of increased professional development and supervisory support.

Women and families who received greater numbers of visits with relatively more parenting content had significantly greater reductions in parenting stress from baseline to follow-up, although the magnitude of effects was modest. Focus on other content areas was also associated with reductions in parenting stress, although effects only approached significance. Reduction in parenting stress is one of the central goals of home visiting programs. Focusing on parenting skills and building parents’ confidence is an important pathway to helping new parents feel less stress as they acclimate to their growing family.

Additionally, our study suggests that for higher-risk families, receiving more home visits may be particularly important to supporting changes in parenting-related attitudes. Families who had greater numbers of risk factors and who also had a greater number of home visits were less likely to endorse the use of corporal punishment compared to high-risk families with fewer home visits. The number of visits received was not associated with changes in parenting attitudes among lower-risk parents. That said, successfully engaging higher-risk families may be particularly important, and they may experience greater benefit from visits than lower-risk families. At the same time, results underscore the importance of providing a sufficient number of home visits in order to achieve desired changes in parenting and other outcomes, a feature of home visiting that has long been recognized but can be challenging to achieve (Gomby et al. 1999; Howard and Brooks-Gunn 2009). Programs would also do well to consistently screen, identify, and enroll families dealing with multiple stressors, and work on creative strategies and schedules to provide visits often and regularly for these women. Given the realities of living with numerous life challenges and the potential difficulties in complying with a “regular” schedule of home visits, designing early engagement strategies that build relationships and trust with families, and providing flexible visit structure options may help increase success in reaching these families.

### Limitations and Future Research

These findings should be considered within the context of the limitations of the study, and within the broader context of study results for home visiting programs nationally. First, generalizability of our findings to all women in home visiting programs is not possible, as the sample included only those who completed surveys after indicating initial interest. It is possible that those who were not included are systematically different in some (unmeasured) ways from those included in the final sample, for example, if the most vulnerable families chose not to participate. Second, the measure of home visiting content, while instructive, included the home visitors’ subjective estimates of the relative emphasis of different topics covered with women and families. Future research is needed to validate this approach, including objective observations of visits and concurrent parent report of visit content.

Further, data collection started later than originally planned which created gaps in home visit logs during the first 90 days of enrollment. As a result, we did not have complete information about content for all visits. Instead, we developed estimates of the *average* time spent in each content area. This approach is inherently limited, as it assumes that home visitors provide roughly the same type of content evenly across home visits from enrollment to family exit. It is possible, however, even likely, that greater amounts of time are spent in early visits on some topics relative to others, a dynamic that could not be reflected in our data. More precise measures of visit content might include tablet-based recording of activity immediately during or after visits or coding based on videotaped visits. Additional research to understand how content changes over time (e.g., greater information/referrals at early visits) would be informative as to whether the type of content provided early versus later facilitates (or impedes) a family’s willingness to engage in continued services.

The delays in start-up may also have reduced the study’s ability to detect changes over time in parenting outcomes. First, some Time 1 parent surveys were sent later than planned, an average of 120 days after a family’s initial enrollment in home visiting, possibly leading to elevated baseline scores. Second, sample sizes in the current study precluded potentially meaningful subgroup analysis (e.g., comparing differences in visit content or outcomes for families with different baseline characteristics). Future research should strive to follow families for a longer period of time. The original design called for 12 months between baseline and follow-up to maximize exposure to program content. However, about half of the sample were enrolled for less than 6 months at the follow-up time point, a short period to be able to reveal meaningful outcome changes. To better explore the relationship of visit and content dosage over time as they relate to positive parenting outcomes, future studies should ideally follow families from enrollment to program completion (up to 3 years in some MIECHV-funded programs).

An important area for future research suggested by these findings includes exploring the relationship of women’s trauma histories to both visit content and outcomes of home visiting. Women are routinely asked to report about multiple areas of interpersonal struggle (e.g., ACEs, depression, intimate partner violence). Looking closely at how programs and visitors may or may not “flex” to accommodate client needs around disclosure of trauma is key. Providing trauma-informed practices implies that those women who disclose significant adverse life events or mental health challenges may benefit from spending more time during visits discussing emotional and mental health issues. Does spending more time in self-care/emotional health content during visits link to improvements in parent mental health functioning or positive parenting practices? Are specific programs or types of visitors better suited to support these women and families? Given the growing awareness of the extent of past trauma and existing struggles for many of the women receiving home visiting services, better understanding of how program content and visit schedules can be tailored to best meet parent needs is a priority for future research.
