# Correction to: What’s Happening During Home Visits? Exploring the Relationship of Home Visiting Content and Dosage to Parenting Outcomes

**DOI:** 10.1007/s10995-018-2627-6

**Published:** 2018-08-22

**Authors:** Peggy Nygren, Beth Green, Katie Winters, Anna Rockhill

**Affiliations:** 1 Regional Research Institute for Human Services, Graduate School of Social Work, Portland State University, Market Center Building, Suite 900,1600 SW 4th Ave, Portland, OR 97201 USA; 2 Center for Improvement of Child and Family Services, Graduate School of Social Work, Portland State University, Market Center Building, Suite 400,1600 SW 4th Ave, Portland, OR 97201 USA

## Correction to: Maternal and Child Health Journal 10.1007/s10995-018-2547-5

The article “What’s Happening During Home Visits? Exploring the Relationship of Home Visiting Content and Dosage to Parenting Outcomes”, written by Peggy Nygren, Beth Green, Katie Winters and Anna Rockhill, was originally published electronically on the publisher’s internet portal (currently SpringerLink) on 13 June 2018 without open access. With the author(s)’ decision to opt for Open Choice the copyright of the article changed on 9 July 2018 to © The Author(s) 2018 and the article is forthwith distributed under the terms of the Creative Commons Attribution 4.0 International License (http://creativecommons.org/licenses/by/4.0/), which permits use, duplication, adaptation, distribution and reproduction in any medium or format, as long as you give appropriate credit to the original author(s) and the source, provide a link to the Creative Commons license and indicate if changes were made.

The original article has been corrected.

